# Influence of Hypothermic Storage Fluids on Mesenchymal Stem Cell Stability: A Comprehensive Review and Personal Experience

**DOI:** 10.3390/cells10051043

**Published:** 2021-04-28

**Authors:** Aneta Ścieżyńska, Marta Soszyńska, Patrycja Szpak, Natalia Krześniak, Jacek Malejczyk, Ilona Kalaszczyńska

**Affiliations:** 1 Department of Histology and Embryology, Medical University of Warsaw, 02-004 Warsaw, Poland; asciezynska@wum.edu.pl (A.Ś.); marta.soszynska@onet.eu (M.S.); pszpak@wum.edu.pl (P.S.); jacek.malejczyk@wum.edu.pl (J.M.); 2 Laboratory of Experimental Immunology, Military Institute of Hygiene and Epidemiology, 01-163 Warsaw, Poland; 3 Department of Plastic Surgery, Medical Centre for Postgraduate Education, 00-416 Warsaw, Poland; natalia.krzesniak@wp.pl; 4 Laboratory for Cell Research and Application, Medical University of Warsaw, 02-097 Warsaw, Poland

**Keywords:** mesenchymal stem cells, cell therapy, preservation solutions, storage media, cell viability, quality control

## Abstract

Mesenchymal stem cells have generated a great deal of interest due to their potential use in regenerative medicine and tissue engineering. Examples illustrating their therapeutic value across various in vivo models are demonstrated in the literature. However, some clinical trials have not proved their therapeutic efficacy, showing that translation into clinical practice is considerably more difficult and discrepancies in clinical protocols can be a source of failure. Among the critical factors which play an important role in MSCs’ therapeutic efficiency are the method of preservation of the stem cell viability and various characteristics during their storage and transportation from the GMP production facility to the patient’s bedside. The cell storage medium should be considered a key factor stabilizing the environment and greatly influencing cell viability and potency and therefore the effectiveness of advanced therapy medicinal product (ATMP) based on MSCs. In this review, we summarize data from 826 publications concerning the effect of the most frequently used cell preservation solutions on MSC potential as cell-based therapeutic medicinal products.

## 1. Introduction

### 1.1. Properties of Mesenchymal Stem Cells

Mesenchymal stem cells or mesenchymal stromal cells (MSCs) are non-hematopoietic, multipotent stem cells that upon stimulation can differentiate into cells of mesodermal, ectodermal, or endodermal lineages [1,2,3,4]. Tri-lineage differentiation potential (osteogenic, chondrogenic and adipogenic), together with the ability to adhere to culture surfaces under standard culture conditions, expression of CD73, CD90, and CD105, and absence of CD45, CD34, CD14, or CD11b, CD79α or CD19, and HLA-DR surface antigens, are part of the standard criteria defining MSCs as set out by the International Society of Cellular Therapy (ISCT) [5]. Discovered initially in the bone marrow [6,7], MSCs may also be isolated from other tissues, e.g., adipose [8], extra-embryonic structures [9,10], or dental tissues [11].

Mesenchymal stem cells have important properties from the perspective of their applicability as medicinal products [12,13]. In the recent past, the dominant hypothesis was that the healing properties of MSCs result from their direct incorporation into the tissue. However, several studies demonstrated that the rate of MSC survival and engraftment seems to be low and transient, and may not explain the therapeutic results achieved by MSCs [14]. On the other hand, other reports confirmed that the regenerative and immunoregulatory activity of MSCs relies on trophic factors that they secrete, including MSC-derived extracellular vesicles (EVs or MSC-EVs), which stimulate resident cells to undertake physiological regenerative processes [15,16,17].

MSCs secrete a variety of proangiogenic, chemotactic, anti-apoptotic, anti-inflammatory, immunomodulating and remodeling soluble factors—platelet-derived growth factor-B (PDGFB), vascular endothelial factor (VEGF), angiopoietin-1 (Ang1), chemokine (C-X-C) ligand (CXCL2, CXCL12), chemokine (C-C) ligand (CCL2, CCL5), interleukin-1 receptor antagonist (IL-1Ra), transforming growth factor beta (TGF-β), stromal-derived factor-1 (SDF1), indoleamine 2,3-dioxygenase (IDO), prostaglandin E2 (PGE2), matrix metalloproteinase-9 (MMP-9), and others [16,18,19].

Due to the properties described above, MSCs may seem to be “doomed to succeed”. However, most of the MSC-based therapies did not progress beyond preclinical or early clinical studies. There is an ongoing debate on the reasons why non-optimal clinical outcomes have been achieved [20,21]. Among others, the following factors could determine the effectiveness of MSCs: (1) the origin of source tissue [22]; (2) donor-to-donor [23], cell-to-cell [24], and passage-to-passage [25] variability; (3) various isolation and expansion methods including different oxygen levels [26] and culture medium composition [27], passage number, [28] preservation method; (4) route of delivery [29] and the number of cells administered [30]; and (5) health condition of the recipient. In an elegant review, Levy and Kuai et al. tackled each of the identified barriers to the implementation of MSC-based therapies [31]. It was shown that problems with diversity in the manufacturing and quality control of MSC-based medicinal products, as described by Mendicino et al., have still not been solved [32]. This applies to tissue sourcing, propagation methods, and in vitro and in vivo product characterization described in investigational new drug (IND) submissions to the U.S. FDA for MSC-based products.

A striking example illustrating the influence of different isolation, culture, and cryopreservation methods was demonstrated by Stroncek et al. in an experiment in which MSCs were manufactured from the same source material [33]. The authors pointed out that “manufacturing of MSCs by five independent centers contributed more to MSC variability than did the source material of the bone marrow” used in the study.

All these differences may lead to significant variations in MSC efficacy and potency. A validated potency assay with defined acceptance criteria secures consistency of quality and ensures that the medicinal product delivered to the patient will bring about the expected results. Due to the nature of cell-based medicinal products (a wide range of mechanisms of action), there is no consensus on any potency assay for MSCs. For now, ISCT minimum criteria [5] and viability verification provide a basis for establishing the quality of MSCs before their therapeutic application. The disadvantage of the current phenotypic classification of MSCs is that the characterized population is heterogeneous and subpopulations with different proliferative and differentiation potential can be distinguished [34,35,36]. This heterogeneity results in non-uniform tri-lineage differentiation potential, as was demonstrated in clonal studies with less than 50% of bone marrow-derived MSC clones possessing the tri-lineage differentiation potential [37].

MSC identity and potency testing are likely to change as more research is conducted to better define properties of MSCs—for example, additional phenotypic criteria including STRO-1, TWIST-1, DERMO-1, GSTT1, CD271 [38,39,40,41], an in vitro model of IL-10 release from blood cells [42], and MSC-mediated inhibition of T-cell activation [43,44]. However, such assays for potency testing of MSCs are not part of routine quality control, and their applicability remains to be determined.

It turns out that the manufacturing and application of cell-based therapies create problems of a different nature from those which are characteristic of chemical or protein-based drugs. Understanding and taming these differences is important for the successful development of the cell-based product formula, its efficient and safe delivery, and application.

### 1.2. Mesenchymal Stem Cells—Their Fate between Manufacturer and Clinic

A distinctive feature of the cell-based medicinal product is its low stability over time, and the maintenance of stability is crucial for the safe and effective implementation of cell-based therapies, as a lot of time can pass between the release from the GMP facility and administration of the product. A significant number of in vitro studies show that cell storage conditions induce changes in cellular morphology, viability, and therapeutic properties [45,46,47]. Consequently, this may result in insufficient therapeutic potency of cell-based medicinal products with a substantially reduced number of live cells. As one of the studies illustrates, over 50% of human MSC-based products derived from the umbilical cord do not meet the criteria of sufficient stability, as their viability at the moment of implantation is below 70% [48].

Unfortunately, at the early stages of cell-based product development, little attention was paid to the ways and means of cell-based medicinal product biopreservation before their application. As for the temperature, it is clear that hypothermic preservation at 2–8 °C is the most reproducible and non-cell-destructive storage system, as it supports higher viability of cells in comparison to those stored at room temperature [49]. Furthermore, although it is certainly most convenient to thaw and directly inject, this practice does not seem to be optimal. Freshly harvested MSCs can be stored for about 6–8 h without affecting their viability and differentiation potential, whereas frozen-thawed cells can be maintained for a maximum of 2 h after thawing before disturbing their viability significantly [50]. There is evidence that MSCs need a recovery phase after cryopreservation, prior to injection, to allow for best in vivo survival and immunomodulatory and therapeutic properties [51,52]. As for the storage solutions, their influence has only recently been taken into consideration, and in addition to well-known crystalloid solutions, media dedicated to the storage of cells-based medicinal products have also appeared on the market.

During cell storage, several metabolic pathways may be affected and cause cell oedema, accumulation of hypoxanthine and xanthine oxidase as well as a breakdown of ion homeostasis due to cell membrane depolarization [53]. Even though MSCs are considered to be more resistant to osmotic and oxidative stresses compared to differentiated cells, vehicle solutions should minimize or prevent those damaging conditions. To reduce cold-induced ionic perturbation, storage media ionic balance needs to be similar to the intracellular milieu and minimize the decrease in cell viability over time [54]. Moreover, a desirable feature of cell storage fluids would be the possibility of their direct administration together with cells. Therefore, due to the lack of dedicated cell preservation solutions, well-known “solutions for infusion” were investigated as storage and transportation solutions for cell-based medicinal products. Their composition mimics human physiological plasma electrolyte concentrations, osmolality and pH, hence they are more similar to the external than internal milieu. Solutions for infusion (also known as intravenous fluids or solutions) are supplemental fluids used in intravenous therapy to restore or maintain normal fluid volume and electrolyte balance and contain compounds that are semipermeable to capillary membranes or freely permeable ions in defined concentrations and can be grouped into colloid or crystalloid solutions, respectively [55]. Albumin suspended in saline may be considered a standard colloidal solution for infusion; however, its limited availability and high cost lead to the implementation of other semisynthetic compounds, e.g., succinylated gelatin, urea-linked gelatin-polygeline, dextran solutions, or hydroxyethyl starch [55]. On the other hand, crystalloids are “balanced” or “physiologic” solutions with multiple different formulations that closely mimic electrolyte composition, osmolality, and pH of human plasma. Implementation of lactate, acetate, gluconate, or malate anions in these solutions provides their additional buffer capacity. Crystalloids are easy to manufacture, cheap and not allergenic, and therefore, compared to colloids, they are frequently used for replacement or maintenance fluid therapy [56]. Normal saline (0.9%), Ringer’s lactated solution and Hartmann’s solution may be considered as the most popular worldwide [57,58]. Furthermore, the novel generation of crystalloids (Ionosteril, Starofundin, Plasma-Lyte) are also implemented as storage solutions, since they more closely resemble human plasma composition [59].

Limited studies on optimization and validation of the transport/storage conditions are available. Normal saline (0.9% NaCl) or phosphate-buffered saline (PBS) are most frequently used; however, the storage time is limited to 6–12 h (own experience). Ringer’s lactated solution supplemented with 1% human serum albumin (HAS) seems to support the viability of Wharton′s jelly MSCs (WJ-MSC) above the 70% threshold for up to 36 h of hypothermic storage [60]. This combination of components seems to be superior to 0.9% NaCl, 5% dextrose, 5% dextrose in sodium chloride, Plasma-Lyte or 1–5% HAS [48]. Upon storage in the mentioned formulations, WJ-MSCs displayed progressive deterioration in viability and adhesion ability, but the immunophenotype and immunosuppressive and differentiation capacities were relatively unaffected. Interestingly, storage of bone marrow-derived MSCs (BM-MSC) in PBS for 24 h also exhibited osteogenic differentiation capability in vitro, as shown by the mineralized matrix formation and alkaline phosphatase activity when cultured in an osteogenic medium [61]. Similarly, BM stored in 4% HSA in 0.9% saline for 18 h showed adhesion to hydroxyapatite tricalcium phosphate osteoconductive biomaterial (HA/β-TCP 3D scaffold) and subsequent in vivo bone formation [62]. In all of the above studies, it was shown that storage at room temperature was conversely related to viability rates. The addition of 6-chromanol derivate (SUL-109) as a preservation agent that protects cells from hypothermia and rewarming damage without affecting their subsequent differentiation capacity seems an interesting solution [63].

Except for the studies listed above, there are limited reports that mention the type and influence of storage/suspension fluids on stability and properties of MSCs used in clinical trials.

In this paper, based on the literature analysis of 826 publications, we demonstrate which suspension solutions are the most commonly used in clinical trials for MSC-based medicinal product preparation. In addition, we recapitulate information regarding the influence of storage solutions on MSC properties. Such knowledge is indispensable to formulate MSC-based medicinal products of the highest quality.

## 2. Materials and Methods

### 2.1. Literature Analysis

**Figure 1:**
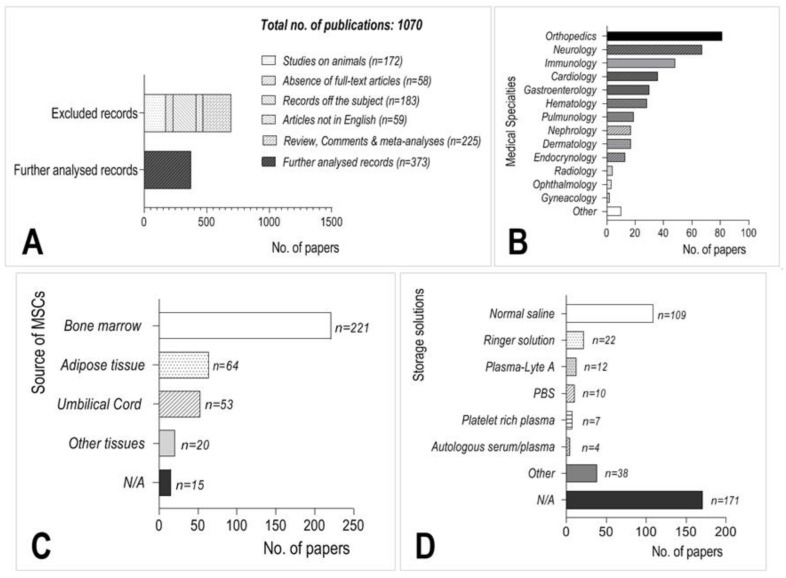
Summary of the results from the literature search. (**A**) Flowchart of the selection process of the analyzed publications. (**B**) The most frequent medical indications in which MSCs were used. (**C**) The most frequent sources of MSCs for cell-based medicinal product preparation. (**D**) The most frequently used storage/suspension solutions in the analyzed literature.

Selected literature (*n* = 826) was retrieved from the National Center for Biotechnology Information Database (https://www.ncbi.nlm.nih.gov/ accessed on 26 February 2021). Search terms were: “Humans” [MeSH]; “Mesenchymal Stem Cell Transplantation” [MAJR]; “Mesenchymal Stem Cell Transplantation methods” [MAJR]; “Treatment Outcome” [MeSH] and “Case Reports” [MeSH] combined in various modifications with Boolean operators “AND” and “OR”. Additional sources of information were retrieved from hand searches of relevant papers not found in the PubMed database and from references of the analyzed publications. Studies performed on cell cultures or animals were excluded from further analysis. Additional analysis was performed for 9 publications in which the viability of MSCs stored in several different media and at least in two time points was validated. Literature analysis was performed in February 2021 (Figure 1A).

### 2.2. Viability Measurement

Adipose tissue was collected from human donors after cosmetic liposuction procedures. The collected tissue would otherwise have been discarded. The procurement of human adipose tissue was approved by the Local Bioethics Committee (approval KB/85/A/2012). The human stromal vascular fraction (SVF) of adipose tissue was isolated using the method originally described by Zuk and co-authors [8]. The detailed procedure used for SVF isolation followed that used in a previous study [64]. The isolated SVF cells were seeded into T75 culture flasks (fibronectin pre-coated, MSC attachment solution, Biological Industries, Israel) at a density of 3 × 10^6^ nucleated cells per flask and cultured at 37 °C and 5% CO_2_ in a humidified atmosphere. The complete culture medium consisted of MSC NutriStem^®^ XF Medium (Biological Industries, Beit Haemek, Israel)—defined, xeno-free, serum-free medium, and 1% antibiotic-antimycotic (Life Technologies, Netherlands). The cells were cultured until reaching approximately 70% confluence. At passage 3, cells were detached from culture plates with Accutase (Thermo Fisher Scientific, Schiphol, Netherlands), washed in PBS and resuspended in 0.9% NaCl; 0.9% NaCl supplemented with glucose to a final concentration of 5% (*w*/*v*); lactated Ringer’s solution (Fresenius Kabi, Warsaw, Poland); Plasmalyte (Baxter, Warsaw, Poland); and HypoThermosol FRS (BioLife Solutions, WA, USA). For cell counting and viability measurement, the cells were labeled with propidium iodide (PI) and counted with an automatic cell counter ADAM MC (NanoEntec, Seoul, Korea) after 12, 24, 36, 48, 72, 96, and 120 h of storage at 4 °C. Cells from 7 donors were tested.

## 3. Results

Based on our literature search, mesenchymal stem cells were used in clinical applications in more than 50 medical conditions and the top three indications were neurology, orthopedics, and immunology (Figure 1B). MSCs were most frequently isolated from bone marrow (Figure 1C); however, adipose tissue and the umbilical cord were also important sources of MSCs for cell-based medicinal products.

Cells used for further clinical analysis were suspended mostly in 0.9% NaCl (normal saline) or Ringer’s solution (70 and 13, respectively) (Figure 1D). Unfortunately, the storage/suspension medium was not specified in almost 200 applications, which significantly hindered the possibility of comparing the outcomes between different clinical trials.

**Figure 2:**
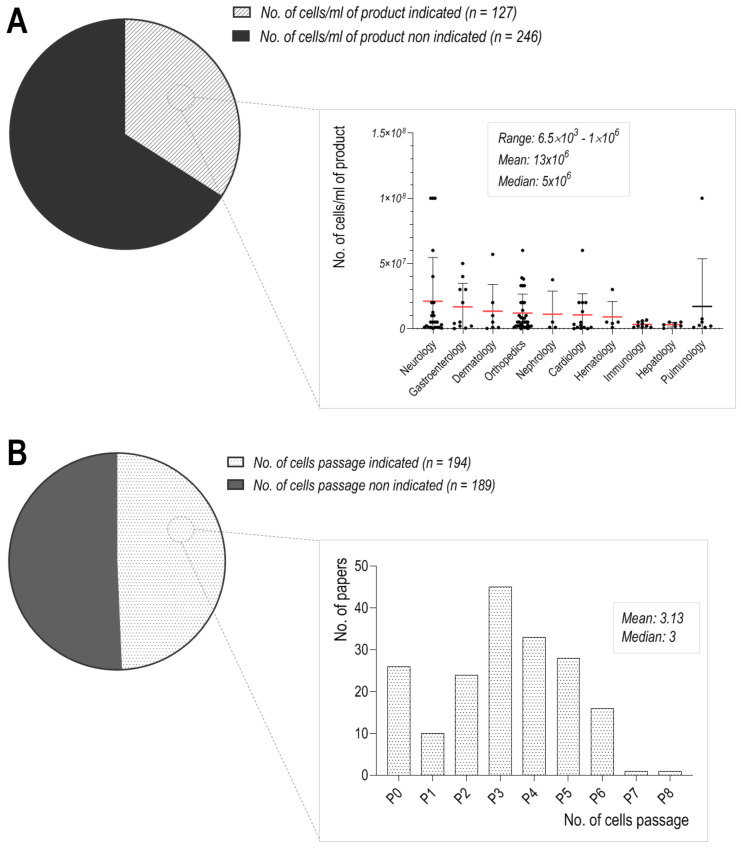
Summary of the results from the literature search. (**A**) The number of cells per milliliter of product, (**B**) Passage number of the MSCs used as a medicinal product.

Apart from that, the number of MSCs suspended in storage/suspension solutions varies greatly, from 10^5^ to 226 × 10^6^, with a median of 4 × 10^6^ of cells. Depending on the medical condition in which the prepared products were used, the quantity of applied MSCs differed significantly (Figure 2A). In the context of medicinal product stability, the number of cells used for final products may affect their viability and lead to different clinical outcomes. Gravitational cellular aggregation or cell clumping was observed in high titer cell suspensions and had adverse consequences in transplantation therapy [65], while at lower MSC concentrations, their migratory capabilities and the overall viability may be elevated [66]. The most concentrated MSC products were used in dermatology, neurology, and gastroenterology (Figure 2A). In the field of dermatology, an average of 48 × 10^6^ MSCs were used for medicinal product preparation; however, the scatter of the results is broad, with a median set at 10^6^ cells. A broad range of MSCs used for clinical trials was also used in the field of orthopedics (from 10^6^ to 39 × 10^6^ of cells). Additionally, in this case, the optimal dose of cells, their co-adjuvants, or appropriate source of harvested cells has not been optimized to date.

It is extremely important to standardize isolation and culture procedures, as the efficacy of MSCs used for the treatment may depend on the employed techniques [67]. MSCs lose their potency during sub-culturing at higher passages, and therefore freshly isolated MSCs have greater engraftment efficiency than the cultured cells. Moreover, culture media used for MSC product preparation may affect cellular performance and commit them into differentiation pathways [68]. In addition to that, the probability of malignant transformation increases during long-term culture [69].

Our analysis revealed high variability between passages of cells used for further clinical applications, which differ greatly from P0 to P8 (Figure 2B), and the overwhelming majority of cell cultures were monolayers. It is well known that the older the cells are, the larger diameter they reach [70]. This may lead to serious vascular obstructions after intravenous delivery [71,72], but it seems that size is not the only factor. Wang et al. demonstrated that with the successive adherent cultures of MSCs, the expression level of integrins increased, which was associated with higher levels of phospho-focal adhesion kinase (FAK) and increased attachment to endothelial cells [73]. Interestingly, MSCs cultured as 3D spheroids are characterized by a significantly smaller size and did not cause detectable infarct in the brain, as demonstrated by Ge et al. [71], and the most plausible reason is that a short period of 3D spheroid culture of MSCs was enough to reduce the size of these cells [74] as well as the expression levels of many integrins [73]. Our analysis of the literature shows that MSCs at passages 3 and 4 were most frequently used in clinical trials. The advantage of earlier passages (1–2) over later passages of MSC application was demonstrated in GVHD patients. In those who were treated with MSCs at passages 1–2, 1-year survival was 75%, in contrast to 21% of patients treated with later passage MSCs (from passages 3–4) [75]. Hence, it is clear that an increasing number of passages affects MSCs’ therapeutic potential.

The performance of MSCs as a therapeutic agent can be further affected by the storage/suspension solution. Various data suggest that solution compositions should address the differences in cell death mechanisms and match specific cell types, customizing them for specific cells and tissue preservation [76]. Therefore, in our analysis of the literature, we focused on the influence of suspension fluids on MSC stability in hypothermic conditions. Hypothermic storage eliminates the need for freezing and thawing equipment and allows the delivery of fresh cells that may be further administered without additional processing steps, which could be another factor introducing high variability and post-thaw loss of isolated cells. Depending on the storage/suspension solution, significant loss of MSC viability may start as early as 2 h after the preparation of the cell suspension [77].

**Figure 3:**
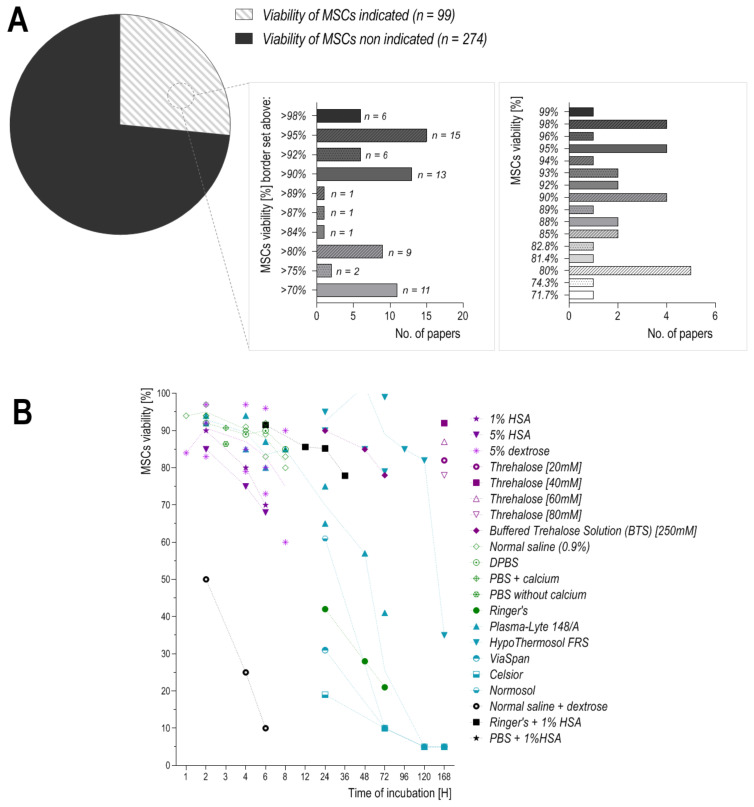
Summary of the results obtained from the literature search. (**A**) MSC viability shown as the minimum level required (left graph) or actual viability at implantation (right graph), (**B**) viability of MSCs incubated in different storage solutions, measured up to 168 h.

Hypothermic storage solutions with high amounts of sodium and chloride ions may lead to cell swelling due to extracellular loss of K^+^ ions and intracellular diffusion of Na^+^ and Cl^−^. Therefore, well-defined media, which mimic an intracellular-like ionic balance with relevant osmolytes, are highly desirable to prepare high-quality MSC-based medicinal products [78]. To date, numerous solutions have been described: from simple physiological saline (0.9% sodium chloride or Ringer’s solution) to more complex solutions containing varying concentrations of sucrose and/or high molecular weight polyethylene glycol, human serum albumin, hydroxyethyl starch, or dextrose in various concentrations [48]. Protein-free and nutrient-poor solutions, even though they minimize the chances of immunological and inflammatory reactions upon delivery of MSCs in situ, may be insufficient to maintain high MSC viability for long periods [65]. To date, the effects of storage/suspension solutions on MSCs have been analyzed only in a few studies. These publications focused mainly on the most commonly used solutions, even though some of them are not suitable for clinical use (i.e., phosphate buffer saline, PBS). Apart from that, cell viability may differ due to the inconsistency of the method used for viability measuring. The use of trypan blue may generate a 5–8% difference between automated and manual techniques, while fluorescent measurement of DNA-binding dye propidium iodide (PI) may result in false positivity [60]. Moreover, differences in viability measurement between the TB exclusion assay and fluorescence-based viability stains can reach as much as 30% [79]. Despite these differences, we searched for publications that evaluate the viability of MSCs which were resuspended in different storage/suspension fluids and their viability was measured at a minimum of two-time points. Moreover, we paid special attention to the information on the specification of the medicinal product; that is, the minimum viability (determined by the manufacturer) required for the ATMP product to be administered. This borderline needs to be determined for every type of cell-based ATMP. Unfortunately, this parameter or actual viability of administered cells was provided in only 36.1% of publications (Figure 3A,B). The requirements for the viability of the final cell-based medicinal product (borderline) are very different—they range from 70% to 98%. It can only be presumed that the authors of the work validated the process and ensured that the given product maintained the expected viability at the time of administration. On the other hand, some authors provided the actual viability of cells at administration—it also ranged from 71.7% to 99%. A medicinal product with a 99% viability will return different clinical results from one with a 70% viability. Therefore, we should strive to narrow the acceptable viability ranges for our cellular medicinal products. One way to achieve this is to administer the product immediately after its manufacturing, and the other is to use such fluids to store cells that will provide an appropriate external environment and prolong their viability.

Most information about storage fluids comes from studies in which the viability of cells stored up to 24 h was determined. Of all the fluids tested after 24 h, HypoThermosol FRS seems to provide cells with the greatest survival rate, reaching 80% even after 120 h of storage.

Ringer’s solution is the second most popular injectable isotonic fluid for medical use that can be directly administered to patients. Lactate is frequently added to Ringers’ solution to reduce its chloride content, which may cause hyperchloremic metabolic acidosis after infusion of large volumes. Lactate Ringer’s solution has a roughly similar composition to plasma and supposedly supports cells more efficiently than normal saline (0.9%) [60]; however, there are no sufficient data to compare the influence of both fluids on MSC viability within the same time (Table 1). The lifespan of cells stored over 24 h in lactated Ringer’s fluid decreases significantly (Table 1). Interestingly, the viability of MSCs after 24 h of storage is over two times greater when cells are suspended in a mixture of 1% human serum albumin (HSA) and Ringer’s solution than in Ringer’s solution alone. Additionally, the viability of MSCs suspended in 1% HSA is greater than in the case of cells suspended at higher (5%) albumin concentrations. However, supplementation of lactated Ringer’s solution with HSA would unfortunately increase the cost of product preparation.

Trehalose may be an interesting colloidal alternative to human serum albumin. It is a non-toxic small disaccharide of glucose, which exhibits strong neuroprotective properties, reduces oxidative stress, and stabilizes the phospholipid bilayers in mammalian cells [45]. Trehalose reduces the need for high permeable cryoprotectant concentrations and effectively improves the survival of MSCs up to as much as 2–3 weeks of storage at 4 °C, without alteration in their morphology and kinetics of growth [80]. High viability of MSCs is observed for trehalose concentrations of 20–40 mM (Table 1); however, more experiments should be performed to confirm these observations.

**Table 1:** Changes in cells viability over time.

		Incubation Period [Hours]														
		Short Period						Long Period								
Storage Media		1	2	3	4	6	8	12	24	36	48	72	96	120	168	Ref
**Colloids**	**1% HSA**	nd.	90% (*n* = 1)	nd.	80% (*n* = 1)	70% (*n* = 1)	nd.	nd.	nd.	nd.	nd.	nd.	nd.	nd.	nd.	[48]
	**5% HSA**	nd.	85% (*n* = 1)	nd.	75% (*n* = 1)	68% (*n* = 1)	nd.	nd.	nd.	nd.	nd.	nd.	nd.	nd.	nd.	[48]
	**5% dextrose**	84% (*n* = 1)	90.7% (*n* = 3)	nd.	87% (*n* = 3)	83% (*n* = 3)	75% (*n* = 2)	nd.	nd.	nd.	nd.	nd.	nd.	nd.	nd.	[48,50,81]
	**Trehalose Solution [20 mM]**	nd.	nd.	nd.	nd.	nd.	nd.	nd.	nd.	nd.	nd.	nd.	nd.	nd.	82% (*n* = 1)	[80]
	**Trehalose Solution [40 mM]**	nd.	nd.	nd.	nd.	nd.	nd.	nd.	nd.	nd.	nd.	nd.	nd.	nd.	92% (*n* = 1)	[80]
	**Trehalose Solution [60 mM]**	nd.	nd.	nd.	nd.	nd.	nd.	nd.	nd.	nd.	nd.	nd.	nd.	nd.	87% (*n* = 1)	[80]
	**Trehalose Solution [80 mM]**	nd.	nd.	nd.	nd.	nd.	nd.	nd.	nd.	nd.	nd.	nd.	nd.	nd.	78% (*n* = 1)	[80]
	**Trehalose Solution [250 mM]**	nd.	nd.	nd.	nd.	nd.	nd.	nd.	90% (*n* = 1)	nd.	85% (*n* = 1)	78% (*n* = 1)	nd.	nd.	nd.	[45]
**Crystalloids**	**Normal saline (0.9%)**	94% (*n* = 1)	95% (*n* = 3)	nd.	90.3% (*n* = 3)	88% (*n* = 3)	81.5% (*n* = 2)	nd.	nd.	nd.	nd.	nd.	nd.	nd.	nd.	[48,50,81]
	**DPBS**	nd.	92% (*n* =1)	nd.	89% (*n* = 1)	90% (*n* =1)	85% (*n* = 1)	nd.	nd.	nd.	nd.	nd.	nd.	nd.	nd.	[50]
	**PBS + calcium**	nd.	nd.	90.7% (*n* = 1)	nd.	nd.	nd.	nd.	nd.	nd.	nd.	nd.	nd.	nd.	nd.	[65]
	**PBS without calcium**	nd.	nd.	86.4% (*n* =1)	nd.	nd.	nd.	nd.	nd.	nd.	nd.	nd.	nd.	nd.	nd.	[65]
	**Ringer’s**	nd.	nd.	nd.	nd.	nd.	nd.	nd.	42% (*n* = 1)	nd.	28% (*n* = 1)	21% (*n* = 1)	nd.	nd.	nd.	[45]
	**Plasma-Lyte 148/A**	nd.	93% (*n* = 2)	nd.	89.5% (*n* = 2)	83.5% (*n* = 2)	85% (*n* = 1)	nd.	70% (*n* = 2)	nd.	57% (*n* = 1)	25.5% (*n* = 2)	nd.	5% (*n* = 1)	5% (*n* = 1)	[45,48,50,78]
	**HypoThermosol FRS**	nd.	nd.	nd.	nd.	nd.	nd.	nd.	92.5% (*n* = 2)	nd.	102% (*n* = 2)	89% (*n* = 2)	85% (*n* = 1)	82% (*n* = 1)	35% (*n* = 1)	[45,78,82]
	**ViaSpan**	nd.	nd.	nd.	nd.	nd.	nd.	nd.	31% (*n* = 1)	nd.	nd.	10% (*n* = 1)	nd.	5% (*n* = 1)	5% (*n* = 1)	[78]
	**Celsior**	nd.	nd.	nd.	nd.	nd.	nd.	nd.	19% (*n* = 1)	nd.	nd.	10% (*n* = 1)	nd.	5% (*n* = 1)	5% (*n* = 1)	[78]
	**Normosol**	nd.	nd.	nd.	nd.	nd.	nd.	nd.	61% (*n* = 1)	nd.	nd.	10% (*n* = 1)	nd.	5% (*n* = 1)	5% (*n* = 1)	[78]
**Crystalloids + Colloids**	**Normal saline + Dextrose**	nd.	50% (*n* = 1)	nd.	25% (*n* = 1)	10% (*n* = 1)	nd.	nd.	nd.	nd.	nd.	nd.	nd.	nd.	nd.	[48]
	**PBS + 1% HSA**	nd.	nd.	nd.	nd.	91.6% (*n* = 1)	nd.	nd.	nd.	nd.	nd.	nd.	nd.	nd.	nd.	[60]
	**Ringer’s + 1% HSA**	nd.	nd.	nd.	nd.	91.5% (*n* = 1)	nd.	85.6% (*n* = 1)	85.2% (*n* = 1)	77.9% (*n* = 1)	nd.	nd.	nd.	nd.	nd.	[60]

Another commonly used saccharide—dextrose at 5% concentration in water or 0.9% normal saline can support the viability of MSCs for 6 to 8 h, respectively as demonstrated by transplantation efficacy of MSCs (Table 1). Contrary to these observations, it was noted that MSC-based cellular medicinal products suspended in a combination of saline or dextrose are characterized by a significant decrease in cell viability and proliferation potency when the period of storage is longer than 2 h [81]. It was the greatest and fastest decrease in cell viability which has been reported in the literature so far (Figure 3B). The detrimental effect of 5% dextrose on stored cells is due to the replicative senescence of MSCs [48]. Interestingly, the cells stored in normal saline itself, in the corresponding 6 h period of storage, showed an almost nine times greater life span. Furthermore, MSCs stored in Plasmalyte temporarily (up to 8 h) sustain a high rate of transplantation efficiency (above 80%), while their viability decreases to 50% after 48 h of storage, and finally dropping to 5% after 7 days [45,54].

Among crystalloids, the best MSC viability after one week of storage was observed in HypoThermosol^®^ FRS suspension. HypoThermosol^®^ FRS is a commercially available storage solution (HTS-FRS, BioLife Solutions, WA, USA) composed of several nonpermeable substances (e.g., sucrose, lactobionate, and mannitol) and has been described as an effective biopreserving solution for hepatocytes, endothelial cells, and neural stem cells [45]. Long-term (up to 4 days) hypothermic storage was reported to result in above 80% cell recovery with their subsequent normal proliferation and marker expression [82]. It was noted that MSCs stored in HypoThermosol^®^ FRS, Plasmalyte, or buffered trehalose solution for 24 or 48 h present greater viability than those stored in Ringer’s solution [45]. Additionally, HTS-FRS maintains an intact actin cytoskeleton and visually unbroken culture monolayer, while diminished cell numbers were observed in Plasmalyte, Normosol, ViaSpan Custodiol, and Celsior [54].

**Figure 4:**
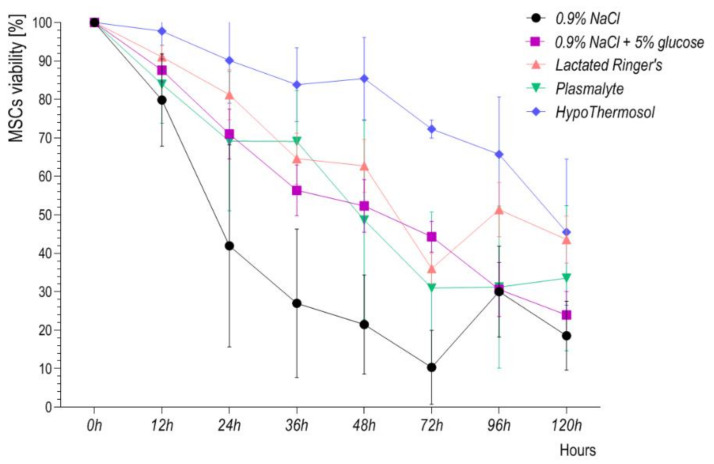
ADSC viability in 0.9% NaCl, 0.9% NaCl with 5% glucose, lactated Ringer’s solution, Plasmalyte, and HypoThermosol FRS monitored over 120 h.

Choosing the right storage fluid is not easy. Most often, we consider those that do not need to be washed out from cellular medicinal products before being administered to the patient. Our own experience confirms poor stability of adipose tissue-derived mesenchymal stem cells (ADSCs) stored in 0.9% NaCl, 0.9% NaCl supplemented with 5% glucose, lactated Ringer’s solution or Plasmalyte (Figure 4). To maintain the minimum 80% viability of cellular ATMP, the product shelf-life should not be longer than 12 h. However, the chosen maximum storage period should be validated, based on data from stability testing. None of the 0.9% NaCl, 0.9% NaCl + 5% glucose, lactated Ringer’s solution or Plasmalyte were designed for cell storage. In contrast, fluids such as HypoThermosol were designed for this purpose, therefore it is not surprising that it ensures the longest maintenance of cell stability (Figure 4).

## 4. Regulatory Framework

Cellular medicinal products fall within the category of advanced therapy medicinal products (ATMP). In general, ATMPs are divided into three subcategories: somatic cell therapy medicinal products (SCTMP), tissue-engineered products (TEP), and gene therapy medicinal products (GTMP). Directive 2004/23/EC of the European Parliament sets out the standards of quality and safety for the donation, procurement, testing, processing, preservation, storage, and distribution of human tissues and cells. Regulation (EC) No 1394/2007 of the European Parliament and of the Council on advanced therapy medicinal products is supplementary to this directive.

The stability validation process is only one of the requirements that have to be fulfilled to implement ATMP into the clinic through one out of three European regulatory pathways: hospital exemption, clinical trial, or centralized marketing authorization.

The principal rationale for stability testing is to ensure the maximum quality and most of all safety of cell-based therapies. Cellular advanced medical/pharmaceutical product stability may be defined as the capability of a dosage form in a particular packaging without losing its potency and other characteristics as detailed in the specifications throughout its “shelf life”.

Detailed results of the stability studies, including information on the analytical procedures used to generate the data and validation of these procedures, must be presented. For the regulatory acceptance of drug products, the stability studies data are a compulsory requirement, and in Europe, these requirements are described in the Commission Directive 2003/63/EC on the Community code relating to medicinal products for human use.

Mesenchymal stem cells (MSCs) represent cell therapy products that under the European Union regulation (EC) No 1394/2007 are classified as advanced therapy medicinal products; hence, their manufacturing is subject to standardized quality systems under Good Manufacturing Practice (GMP) regulation. Available guidelines for ATMPs were designed on the basis of rules relating to conventional medicinal products. However, conventional medicinal products are manufactured with chemical and physical methods, and their characteristic feature is the availability of consistent raw materials and/or active pharmaceutical ingredients, excipients, and a high level of consistency and stability of the finished product. Unlike these products, the manufacture of cellular medicinal products involves biological processes and materials, such as extraction from tissues, cell culture/expansion, preservation. These biological processes may display intrinsic variability, so the end product will have different parameters in each case, such as stability. Due to these differences and requirements for tailored standards for advanced therapy medicinal products, only recently, Annex 2 of GMP was replaced by the Commission guideline on Good Manufacturing Practice for Advanced Therapy Medicinal Products. This guideline has been operational since 22 May 2018.

Another source of recommendations on the design and evaluation of stability tests is presented in a series of guidelines issued by the International Council for Harmonisation of Technical Requirements for Pharmaceuticals for Human Use (ICH). In general, ICH Q1A (R2) and ICH Q5C provide general considerations and set out paths for designing stability testing protocols. Although they cover only chemicals and a range of products of biological origin such as cytokines, antibodies, growth factors, and vaccines, they are a useful foundation for cell-based medicinal product stability testing.

Stability testing at the storage temperature is also one of the points in the guideline of the European Directorate for the Quality of Medicines & HealthCare (EDQM)—“Guide to the quality and safety of TISSUES AND CELLS for human application”. EDQM is a directorate of the Council of Europe responsible for compliance with the quality requirements of the European Pharmacopoeia. In Chapter 9.2.10 “Expiry date”, we can find indications regarding the factors which should be taken into account when determining the storage time—for instance, possible deterioration of the required properties of tissues and cells, expiry of storage solutions, and stability at the storage temperature.

The basic parameter for stability testing of cellular medicinal products is cell viability. Even though quality controls of ATMPs are generally poorly represented and non-compendial, the cell count and viability tests are described in European Pharmacopoeia 10.0, Chapter 2.7.29. “Nucleated cell count and viability” [83].

Finally, studies performed under stress conditions may be useful in establishing whether accidental exposure to conditions other than those proposed (e.g., during transportation) are detrimental to the product. In this regard, ISO 21973:2020 Biotechnology—“General requirements for transportation of cells for therapeutic use” could be helpful. It specifies general requirements and evaluates several aspects to consider the context of transportation of cells for therapeutic use, including storage during transportation between, for example, the manufacturer and the clinic.

As there is an increasing interest in cell-based therapies, we hope for more precise and tailored cellular medicinal product guidelines.

## 5. Study Limitation

As pointed out in the introduction, there is no consensus on any potency assay for MSCs and, at the same time, comprehensive reporting of methodology and biologic characteristics in clinical studies. Phenotypic classification and viability of MSCs are relevant parameters to be evaluated before MSC transplantation, but even more important would be the analysis of proliferation and differentiation potential of MSCs after significant manipulations such as in vitro expansion, condition, and time of storage. In publications from clinical trials indexed in our search, such information was not provided, even though it is vital in order to draw conclusions on the efficacy of cell-based therapies and is also important to relate the results to those described in other publications. Efforts have been made to improve standardization and transparency when describing cell therapies [84]. The proposed DOSES tool provides sufficient information to enable rapid indication of core attributes of the cell-based medicinal product such as donor (D), organ tissue (O), separation method (S), cell characteristics (E), and site of delivery (S). However, the DOSES tool was not designed to provide an exhaustive description of all cell attributes that may influence the efficacy of MSCs. Murray and co-authors presented the position of researchers, and 97% of them agreed that “regulators, societies, and funding bodies should make the reporting of the above tool mandatory when any product involving cell therapies is discussed, and journals should mandate the disclosure of these critical factors when authors describe a cell therapy”. We strongly agree with this statement and we call for its enforcement.

## 6. Conclusions

Prior to clinical application, an MSC-based medicinal product needs to be stored in an appropriate vehicle solution which protects against damaging factors so that the highest cell viability, proliferation, identity, and engraftment capability are maintained. MSC-based medicinal products are tested in many different clinical indications and their effectiveness may depend on the specific properties of the MSCs—proangiogenic, chemotactic, anti-apoptotic, anti-inflammatory or immunomodulating. In turn, these properties may be dependent on many factors including storage solutions. On the basis of the available and still insufficient data, we refrain from giving the reader universal guidelines that should be followed when choosing a storage solution for MSCs intended as medicinal products. Ultimately, regulatory authorities will require validation of a panel of assays used to confirm the identity, potency, and stability of the MSC-based product. Thus, our summary of available studies demonstrates that different storage solutions and the optimal time of storage should be rigorously tested to find the most satisfactory results and establish stability and efficacy profiles for the cell-based medicinal product. It is disappointing that only a quarter of clinical reports provide data on MSC-based medicinal product viability. This also applies to other manufacturing parameters. Hence, we appeal to the manufacturers and scientific community to share information on the important aspects of MSC-based medicinal product manufacturing—only in this way will it be possible to develop universal guidelines ensuring the preparation of medicinal products of the highest quality and safety.

## Data Availability

All relevant data are included within the manuscript. The raw data are available on request from the corresponding author.
